# Case Report: Metastatic Bronchopulmonary Carcinoid Tumor to the Pineal Region

**DOI:** 10.3389/fendo.2021.623756

**Published:** 2021-03-31

**Authors:** Joshua A. Cuoco, Michael W. Kortz, Edwin McCray, Evin L. Guilliams, Christopher M. Busch, Cara M. Rogers, Robert W. Jarrett, Sandeep Mittal

**Affiliations:** ^1^ Department of Neurosurgery, Carilion Clinic Neurosurgery, Roanoke, VA, United States; ^2^ Virginia Tech Carilion School of Medicine, Roanoke, VA, United States; ^3^ School of Neuroscience, Virginia Tech, Blacksburg, VA, United States; ^4^ Department of Neurosurgery, University of Colorado, Aurora, CO, United States; ^5^ College of Osteopathic Medicine, Kansas City University, Kansas City, MO, United States; ^6^ Department of Neurosurgery, Duke University, Durham, NC, United States; ^7^ Carilion Clinic Pathology and Dominion Pathology Associates, Roanoke, VA, United States; ^8^ Fralin Biomedical Research Institute, Roanoke, VA, United States

**Keywords:** metastatic disease, neuroendocrine tumor, lung carcinoid, pineal gland, brain metastasis, CNS tumor

## Abstract

Intracranial spread of a systemic malignancy is common in advanced staged cancers; however, metastasis specifically to the pineal gland is a relatively rare occurrence. A number of primary lesions have been reported to metastasize to the pineal gland, the most common of which is lung. However, metastasis of a bronchial neuroendocrine tumor to the pineal gland is a seldom-reported entity. Here, we present a 53-year-old female who presented with worsening headaches and drowsiness. MRI brain revealed a heterogeneously enhancing partially cystic mass in the pineal region. The patient had an extensive oncologic history consisting of remote stage IIA invasive breast ductal carcinoma as well as a more recently diagnosed atypical bronchopulmonary neuroendocrine tumor with lymph node metastases. She underwent microsurgical volumetric resection of the large pineal mass and a gross total removal of the tumor was achieved. Histopathology confirmed a metastatic tumor of neuroendocrine origin and the immunohistochemical profile was identical to the primary bronchopulmonary carcinoid tumor. Eight weeks after surgery, she underwent stereotactic radiosurgical treatment to the resection cavity. At 1-year follow-up, the patient remains clinically stable without any new focal neurological deficits and without any evidence of residual or recurrent disease on postoperative MRI. Metastatic neuroendocrine tumors should be considered in the differential diagnosis of pineal region tumors and aggressive surgical resection should be considered in selected patients. Gross total tumor resection may afford excellent local disease control. We discuss the relevant literature on neuroendocrine tumors and current treatment strategies for intracranial metastases of neuroendocrine origin.

## Background

The brain parenchyma is a common site for the dissemination of metastatic disease; however, metastasis to the pineal region is very rare with a reported incidence of 0.4–3.8% of all intracranial metastases (1, 2). Since Forster’s original description of the first case in 1858 (3), various primary malignant tumors have been reported to spread to the pineal region including esophageal, stomach, liver, colon, pancreas, kidney, bladder, prostate, thyroid, breast, melanoma, myeloma, and leukemia (4–6). However, there is only one case reported in the literature of a bronchial neuroendocrine tumor with metastasis to the pineal gland (7).

Neuroendocrine tumors constitute a heterogeneous group of malignancies that originate from neuroendocrine cells throughout the body, most commonly arising from the gastrointestinal tract, tracheobronchial tree, and bladder (8, 9). Complete surgical resection of the primary tumor as well as any metastatic deposits with a curative intent is often recommended in patients with limited extent of metastatic disease (8). However, there is a lack of consensus with respect to standard-of-care for unresectable advanced primary tumors or diffuse metastatic disease given a lack of prospective trials (8).

Here, we present a patient with a pineal region metastasis from a known primary lung carcinoid tumor. We discuss the relevant literature on the neuroendocrine tumors and current treatment strategies for intracranial metastases of neuroendocrine origin. To our knowledge, this is the second reported case in the literature.

## Case Presentation

### History and Physical Examination

A 53-year-old female presented to our emergency department with 3 weeks of progressively worsening headaches and drowsiness. No focal neurological deficits were present on examination. Relevant history included stage IIA invasive breast ductal carcinoma treated with left mastectomy and chemotherapy two decades prior. Moreover, 2 years prior to presentation, she was diagnosed with atypical bronchopulmonary carcinoid tumor with metastasis to level 5 lymph node and level 6A lymph node with extracapsular extension (T2aN2M0). She initially underwent treatment with octreotide and lobectomy followed by four cycles of adjuvant cisplatin and etoposide 2 years prior to presentation. Given her progressive neurologic symptomatology and history of malignancy, imaging was obtained to rule-out intracranial pathology.

### Neuroimaging Findings

**Figure 1:**
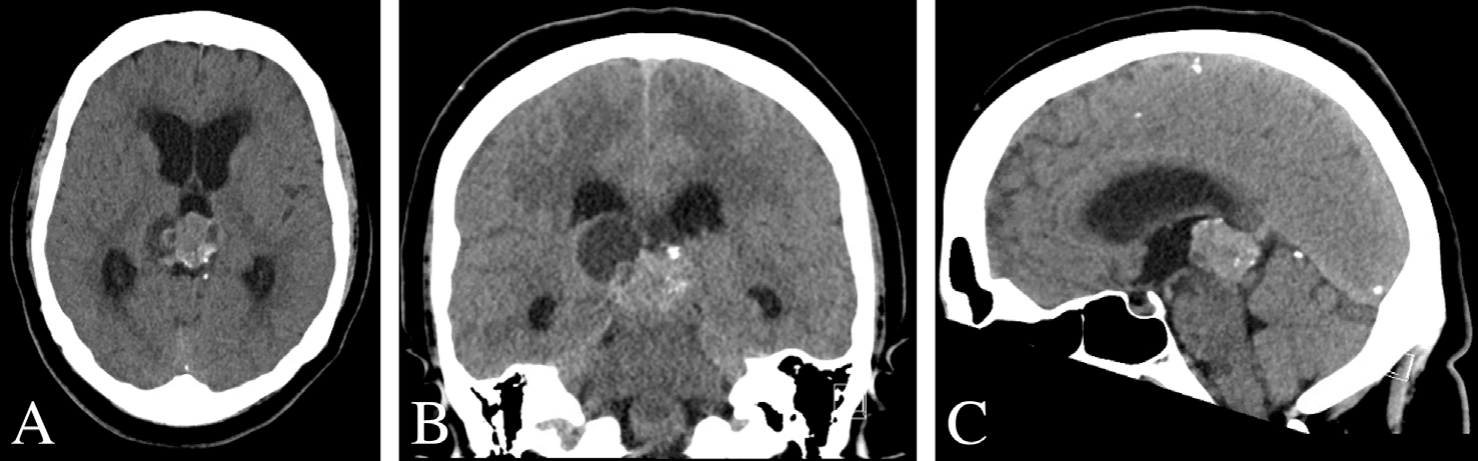
Pre-operative CT scan of the brain. **(A–C)** Non-contrast CT imaging demonstrating a heterogeneous mass with cystic features and calcification in pineal region causing obstructive hydrocephalus.

**Figure 2:**
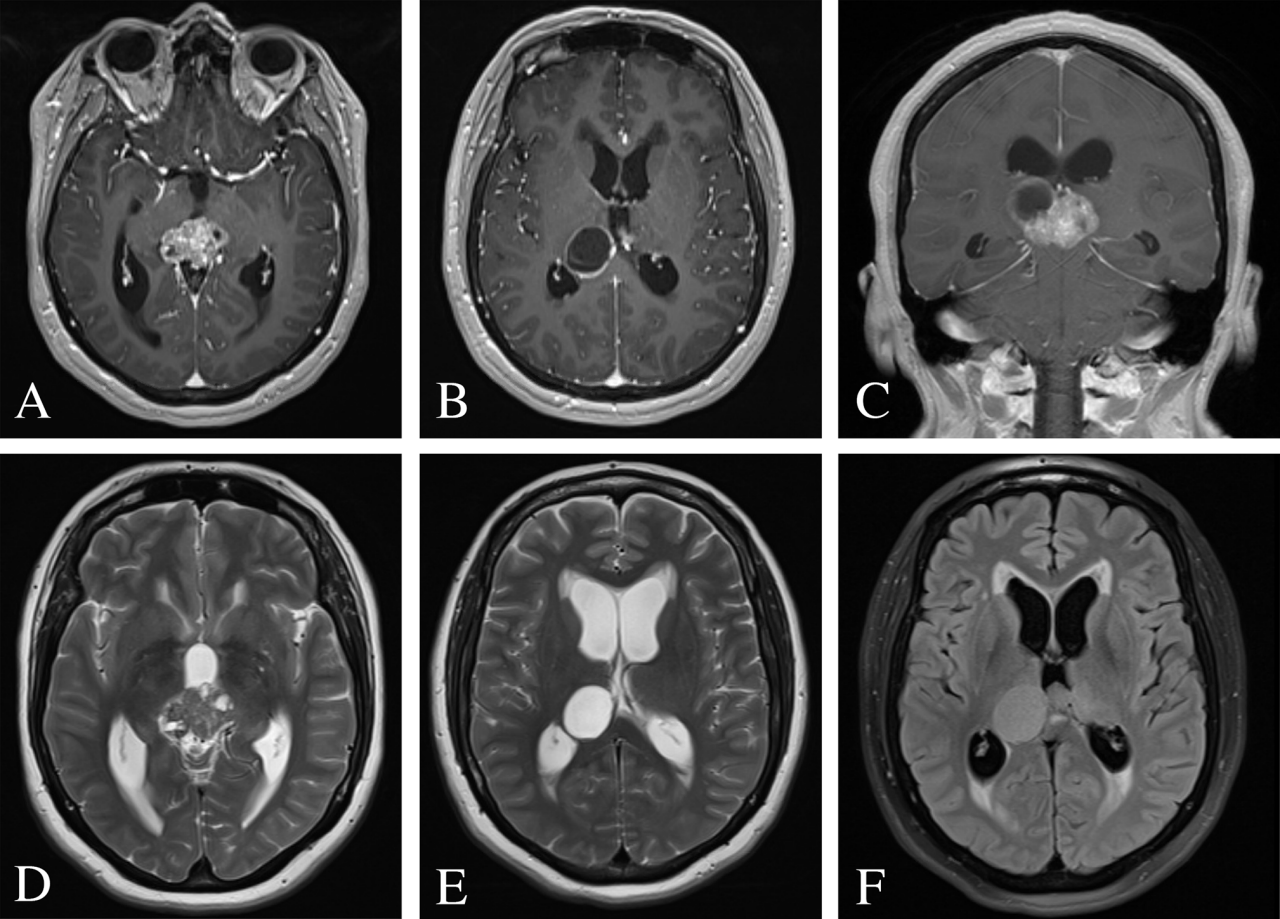
Pre-operative MRI of the brain. **(A–C)** Post-contrast T1-weighted images demonstrating a heterogeneously enhancing 3.9 × 2.6 × 3.1 cm mixed cystic and solid pineal mass and consequential obstructive hydrocephalus. **(D, E)** T2-weighted images revealed a hyperintense cystic lesion and hypointense solid lesion with mass effect and compression of the cerebral aqueduct resulting in supratentorial ventricular dilatation and periventricular white matter signal abnormality. **(F)** FLAIR image demonstrated periventricular transependymal flow of cerebrospinal fluid indicative of acute hydrocephalus.

Computed tomography of the head demonstrated a hyperdense, partially cystic pineal region lesion with areas of calcification with supratentorial ventricular dilatation (**Figure 1**). Magnetic resonance imaging of the brain revealed a heterogeneously enhancing 3.9 × 2.6 × 3.1 cm mixed cystic and solid pineal mass extending into the posterior third ventricle and associated obstructive hydrocephalus (**Figure 2**). Metastatic work-up revealed a stable systemic disease burden.

### Neurosurgical Management and Postoperative Imaging

The patient was started on dexamethasone and medically optimized. Given her relatively young age, symptomatic obstructive hydrocephalus, and need for tissue diagnosis as well as local disease control, neurosurgical resection of the pineal region mass was recommended.

A stereotactic right parietal craniotomy with posterior interhemispheric transcallosal approach was utilized for volumetric resection of the large pineal mass. The patient tolerated the procedure well and without complication. Post-operatively, she exhibited a protracted course with transient neurologic symptomatology (i.e., mildly impaired short-term memory, wide-based gait), which subsequently resolved slowly over 3 months.

**Figure 3:**
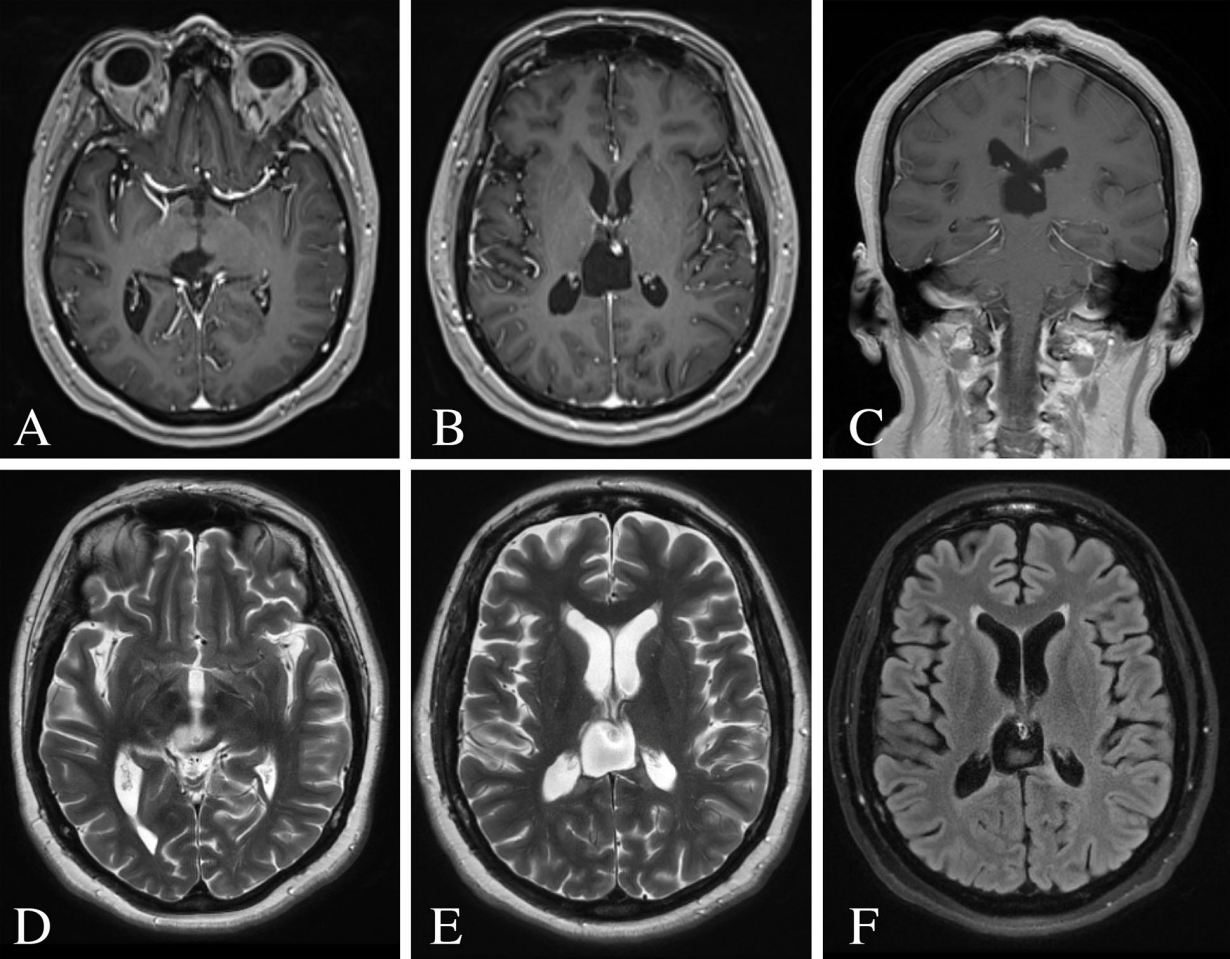
Post-operative MRI of the brain 12 months following surgery. **(A–C)** Post-contrast T1-weighted images demonstrating no evidence of residual or recurrent disease. **(D, E)** T2-weighted images showing resolution of the ventricular dilatation and flow voids from the internal cerebral veins. **(F)** FLAIR image showing minimal hyperintense signal surrounding the surgical resection cavity.

The patient underwent Cyberknife radiosurgical treatment to the resection cavity 8 weeks after surgery. A total of 27 Gy were delivered in three fractions using a total of 126 beams. Repeat MRI brain at 6-months and 1-year revealed no evidence of residual or recurrent disease (**Figure 3**).

### Histopathological Findings

**Figure 4:**
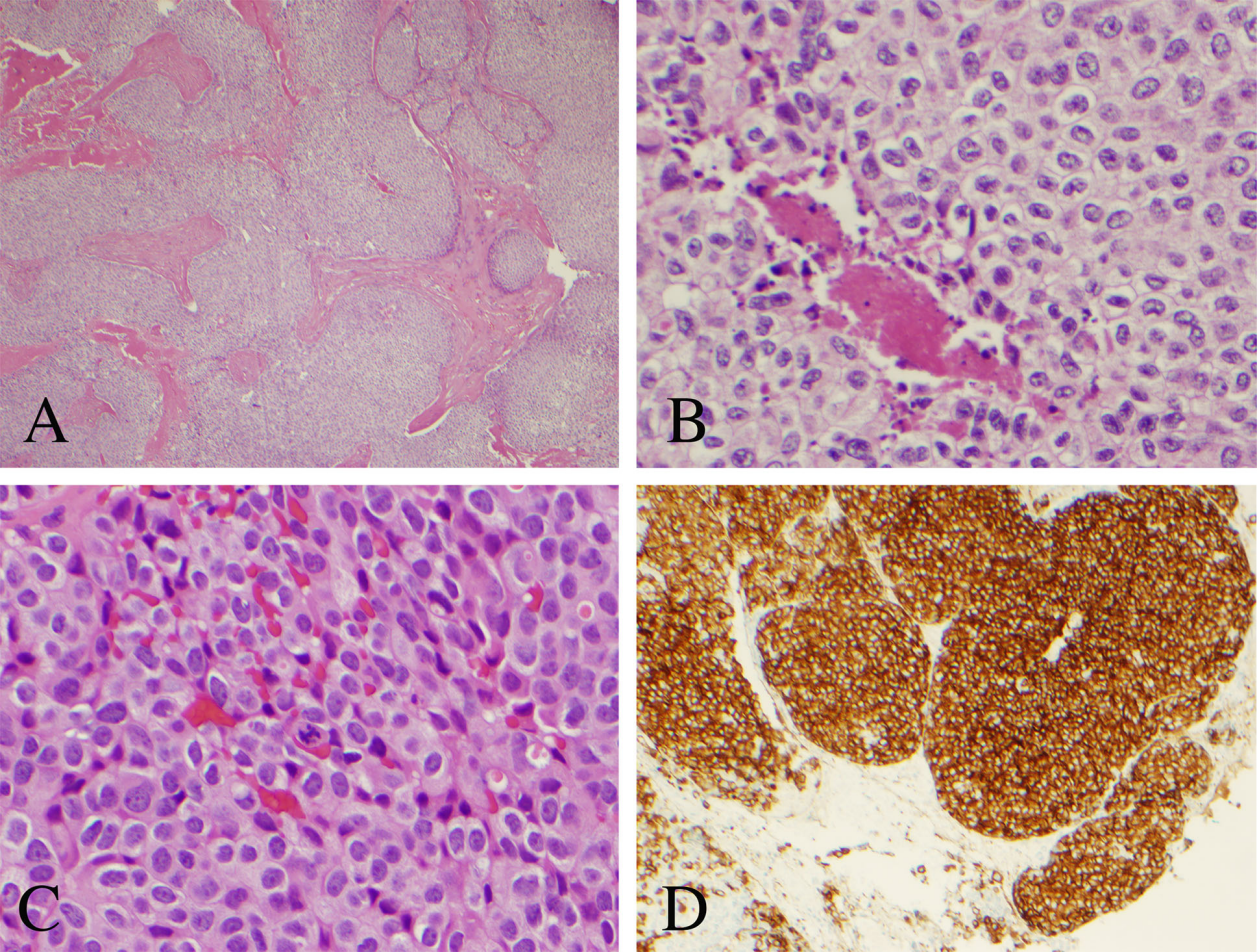
Histopathological analysis of the resected pineal lesion. **(A)** Hematoxylin and eosin (H&E) stain with nested architecture and sheets of cells (original magnification, 40×). **(B)** H&E stain with sheets of cells with speckled chromatin and indistinct to small nucleoli with multifocal necrosis (original magnification, 200×). **(C)** H&E stain with mitotic figure (original magnification, 400×). **(D)** Strong immunostaining of tumor cells with synaptophysin (original magnification, 100×).

**Figure 5:**
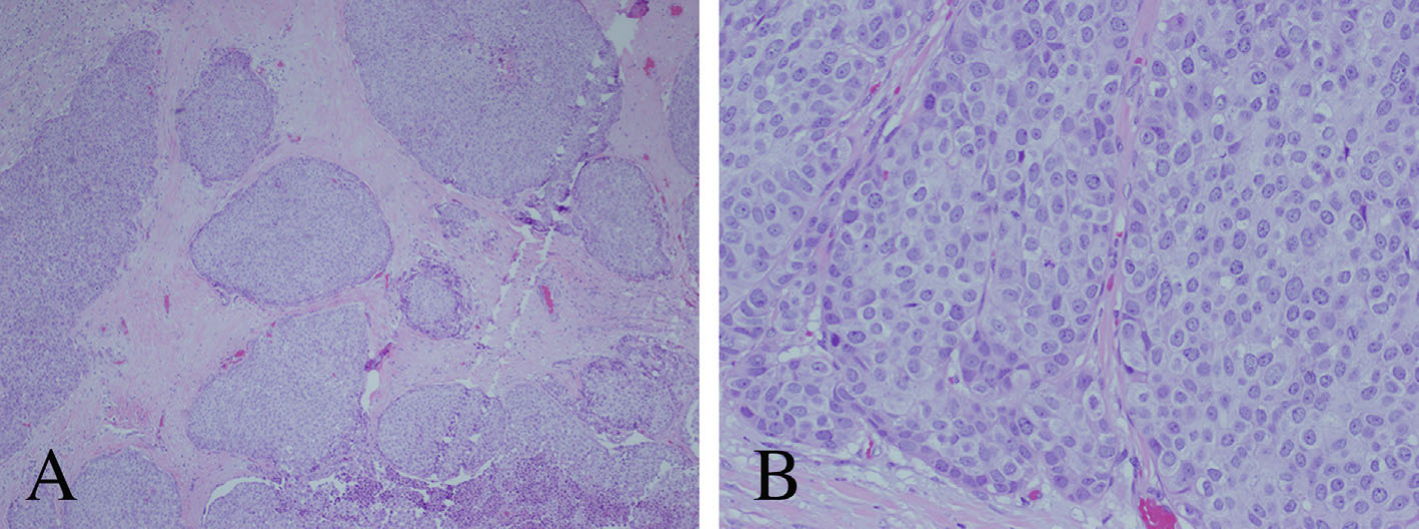
Histopathological analysis of the primary atypical bronchopulmonary carcinoid tumor. **(A)** Hematoxylin and eosin (H&E) stain with nested architecture and sheets of cells (original magnification, 40×). **(B)** H&E stain with sheets of cells with speckled chromatin and indistinct to small nucleoli (original magnification, 200×).

Histopathology was consistent with metastatic atypical carcinoid tumor of pulmonary origin (**Figure 4**). Routine hematoxylin and eosin (H&E) stained sections demonstrated nests and sheets of cells with speckled chromatin and indistinct to small nucleoli. Thick fibrous bands separated the tumor cell nests. The morphologic appearance was identical to that of the primary lung tumor. Mitoses were present less than 10 per 2 square millimeters. Homer Wright rosettes were not seen. The tumor cells were strongly and diffusely reactive with synaptophysin and neuron specific enolase (NSE) immunohistochemical stains. Cytokeratin AE1/AE3 immunostain was strongly and diffusely reactive helping to exclude a primary pineal tumor. Moreover, glial fibrillary acidic protein (GFAP) and S100 immunostains were completely negative, further helping to exclude a primary pineal tumor which can have similar morphology. The Ki-67 proliferative index was 15%, slightly higher than the 7% labeling index noted in the original bronchopulmonary lesion. Comparatively, tumor morphology of the intracranial metastasis was identical to that of the known atypical bronchopulmonary carcinoid lesion diagnosed 2 years prior (**Figure 5**). The primary atypical bronchopulmonary carcinoid lesion stained positive for synaptophysin, CAM5.2, and cytokeratin AE1/AE3. Additional stains of the primary lesion including CK 5/6, CK7, CK20, p40, TTF-1, Napsin-A, and BRST-2 were negative. Necrosis was noted on histopathology and a mitoses count of no more than one per 2 square millimeters.

## Discussion

The pineal gland is a rare location for the spread of systemic metastasis. In fact, metastasis to this neuroendocrine secretory circumventricular organ has a reported incidence of 0.4–3.8% of all intracranial metastases (1, 2). Although lung cancer is the most common source of disease dissemination to the pineal gland, or epiphysis cerebri, various malignant tumors have been reported to spread to this site including esophageal, stomach, liver, colon, pancreas, kidney, bladder, prostate, thyroid, breast, melanoma, myeloma, and leukemia (4–6, 10). There is only one other report in the literature describing pineal gland metastasis from a bronchopulmonary neuroendocrine tumor (7).

Carcinoid tumors are a rare, diverse group of neoplasms that arise from endodermal precursor cells (11). These lesions primarily originate from the gastrointestinal tract and bronchopulmonary system in 54.5 and 30.1% of cases, respectively; nevertheless, they may also arise from a multitude of other organs such as the thymus, pancreas, liver, uterus, ovary, testes, bladder, and rectum (11, 12). Bronchopulmonary neuroendocrine tumors, arising from pulmonary neuroendocrine cells reside as individual cells or as clusters of cells, account for approximately 25% of all primary lung neoplasms (8). These lesions are classified into four distinct subtypes including: (i) well-differentiated, low-grade typical carcinoids; (ii) well-differentiated, intermediate-grade atypical carcinoids; (iii) poorly differentiated, high-grade large cell neuroendocrine carcinoma; and (iv) poorly differentiated, high-grade small cell lung cancer (8, 9). Although typical and atypical carcinoids are categorized as low- and intermediate-grade tumors, respectively, these tumors are capable of regional lymph node metastasis as well as spread to distant sites (8, 13–15). Indeed, 5–20% of typical carcinoids and 30–40% of atypical carcinoids are known metastasize. The most common sites of disease dissemination include regional lymph nodes, lung, liver, and bones (8). Brain metastases are an exceptionally rare finding in patients with neuroendocrine tumors with <5% of patients exhibiting intracranial involvement (16, 17). Furthermore, only 1.4% of metastatic brain tumors are of neuroendocrine origin, the majority of which derive from the lung (16, 17). Nevertheless, brain metastases portend a poor prognosis with a median overall survival of 8 months following initial diagnosis of intracranial dissemination (18). However, the most common cause of mortality in patients with intracranial involvement is attributable to the progression of systemic disease rather than sequelae associated with central nervous system dysfunction (11, 16–18).

Given the rarity of intracranial involvement of neuroendocrine tumors, there are no randomized or prospective data to base definitive treatment recommendations. Variable clinical outcomes have been reported in patients treated with neurosurgical resection, stereotactic radiosurgery, whole-brain radiotherapy (WBRT), or a combined treatment approach of intracranial carcinoid metastases (11, 16, 19, 20). Schupak and Wallner assessed treatment response of external-beam radiation therapy in 44 patients with metastatic or unresectable carcinoid tumors including eight patients with brain metastases (19). The authors found that none of the patients with brain metastases exhibited progression of their intracranial disease after radiation therapy with a median dose of 3,300 cGy. Rather, all eight patients ultimately died due to progression of systemic disease (19). Hlatky et al. analyzed treatment modalities and correlates of survival in 24 patients with brain metastases of carcinoid origin (16). The metastases were treated with WBRT alone in seven patients, surgical resection in 12 patients of which seven received adjuvant WBRT (16). The longest median survival (3.2 years) was observed in the cohort that underwent surgical resection plus adjuvant WBRT (16). Mallory and coworkers evaluated 15 patients with primary carcinoid tumors and intracranial metastases who were treated with either surgical resection (n = 12), stereotactic radiosurgery (n = 2), or WBRT (n = 1) (11). The cohort that underwent surgical resection as the primary treatment modality demonstrated longer progression-free intervals, yet this failed to reach statistical significance (p = 0.095) (9). Krug et al. retrospectively analyzed the clinicopathologic factors and outcome data in a cohort of 51 patients with neuroendocrine tumors with associated brain metastases (20). The authors found no significant difference in median overall survival in patients with brain metastases regardless of intervention. Median overall survival times for WBRT alone, surgical resection plus radiation, or observation were 8 months, 7 months, and 18 months, respectively (p = 0.72) (20). These data have begun to lay the groundwork for optimal treatment modalities for intracranial metastases of carcinoid origin; however, further prospective and randomized trials are necessary to define treatment guidelines.

Although carcinoid tumors have been reported to metastasize to various supratentorial and infratentorial locations, dissemination to the pineal gland is an exceedingly rare phenomenon with only one other report to date in the current literature (7). Grozinsky-Glasberg et al. described a 71-year-old male with brochopulmonary neuroendocrine tumor (unspecified subtype) metastasis to the pineal gland 10 years after initial diagnosis of the primary tumor (7). Endoscopic biopsy of the pineal lesion confirmed a diagnosis of bronchopulmonary neuroendocrine tumor. Of note, the intracranial metastasis exhibited a lower Ki-67 proliferation rate than that of the primary lesion (8 *vs* 20%) confirming heterogeneity of proliferation rates between primary and metastatic cells (7). The authors deemed the lesion inaccessible to surgical resection and instead referred for radiosurgery (unspecified type or dose) (7). The tumor regressed on follow-up imaging although no details were provided regarding follow-up neuroimaging findings or clinical status post-radiation.

In the present case, our patient was diagnosed with atypical bronchopulmonary carcinoid with regional lymph nodes metastasis 2 years prior to presentation. We pursued aggressive neurosurgical resection of the lesion with the intent of gross total resection followed by adjuvant stereotactic radiosurgery to maximize local disease control. One year from the initial operation, she remains clinically stable and follow-up MRI demonstrated no evidence of disease residual intracranially.

Pineal gland lesions are arguably one of the most challenging locations within the intracranial compartment to achieve gross total resection. However, as described herein, aggressive neurosurgical intervention upfront afforded local disease control intracranially at the cost of transient neurologic symptomatology that resolved in the following months. To our knowledge, the present case is first to describe successful neurosurgical resection of a pineal gland lesion of bronchopulmonary neuroendocrine origin. This case highlights a proof-of-concept that aggressive surgical resection of pineal region neuroendocrine metastasis in experienced hands should be considered in the appropriate patient population as gross total resection may afford excellent long-term local disease control.

## Conclusions

We describe a unique case of bronchopulmonary neuroendocrine tumor metastasis to the pineal gland and our treatment approach. The present case demonstrates that neuroendocrine tumor metastasis should be considered in the differential diagnosis of pineal gland lesions. Although there are no standardized treatment guidelines for brain metastases of neuroendocrine origin, with each reported case we obtain a better understanding of the natural history and optimal treatment approach for these rare lesions. An aggressive management approach, including microsurgical resection of pineal region neuroendocrine metastasis, should be considered in carefully selected patients and can provide excellent long-term outcomes.

## Data Availability Statement

The original contributions presented in the study are included in the article/supplementary material. Further inquiries can be directed to the corresponding author.

## Ethics Statement

Ethical review and approval was not required for the study on human participants in accordance with the local legislation and institutional requirements. The patients/participants provided their written informed consent to participate in this study. Written informed consent was obtained from the individual(s) for the publication of any potentially identifiable images or data included in this article.

## Author Contributions

JC and MK are the primary authors of the manuscript. JC, MK, EM, EG, CB, CR, RJ, and SM provided substantial contributions to the conception and design of the manuscript. JC, MK, EM, EG, CB, CR, RJ, and SM agreed to be accountable for all aspects of the work ensuring that questions related to the accuracy or integrity of any part of the work are investigated and resolved. All authors contributed to the article and approved the submitted version.

## Funding

Funding was provided by Carilion Clinic Neurosurgery and the Fralin Biomedical Research Institute at Virginia Tech Carilion School of Medicine.

## Conflict of Interest

The authors declare that the research was conducted in the absence of any commercial or financial relationships that could be construed as a potential conflict of interest.

## Abbreviations

WBRT, whole-brain radiotherapy.
